# Heparanase (HPSE) genetic variants as prognostic indicators in ovarian cancer: evidence from discovery and validation cohorts

**DOI:** 10.1007/s11033-026-11891-y

**Published:** 2026-05-05

**Authors:** Inês Guerra de Melo, Valéria Tavares, Joana Savva-Bordalo, Mariana Rei, Joana Liz-Pimenta, Deolinda Pereira, Rui Medeiros

**Affiliations:** 1 Molecular Oncology and Viral Pathology Group, Department of Pathology and Laboratory Medicine/RISE - Associate Laboratory (Health Research Network), IPO Porto Research Centre (CI-IPOP), Portuguese Oncology Institute of Porto (IPO Porto), Porto Comprehensive Cancer Centre Raquel Seruca (Porto.CCC), Porto, 4200-072 Portugal; 2 Faculty of Medicine, University of Porto (FMUP), Porto, 4200-072 Portugal; 3 Research Department, Portuguese League Against Cancer (NRNorte), Porto, 4200-172 Portugal; 4 Instituto de Ciências Biomédicas Abel Salazar (ICBAS), University of Porto, Porto, 4050-313 Portugal; 5 School of Medicine and Biomedical Sciences (EMCB), Fernando Pessoa University, Gondomar, 4420-096 Portugal; 6 Department of Medical Oncology, Portuguese Oncology Institute of Porto (IPO Porto), Porto, 4200-072 Portugal; 7 Department of Gynaecology, Portuguese Oncology Institute of Porto (IPO Porto), Porto, 4200-072 Portugal; 8 Department of Medical Oncology, Centro Hospitalar de Trás-os-Montes e Alto Douro (CHTMAD), Vila Real, 5000-508 Portugal; 9 Faculty of Health Sciences, Fernando Pessoa University, Porto, 4200-150 Portugal

**Keywords:** Ovarian Neoplasms, Endothelium, Heparanase, Genetic Variation, Gene Expression Regulation, Prognosis

## Abstract

**Background:**

Heparanase uniquely cleaves heparan sulfate, the main component of the outer layer of endothelial cell plasma membranes, promoting tumour invasion and dissemination. However, it can also enhance tumour immune surveillance and clearance. heparanase’s versatility extends to pro-thrombotic properties, such as the promotion of tissue factor release. Interestingly, elevated heparanase levels have been found in ovarian cancer (OC), which has a notably high incidence of venous thrombosis. Previously, single-nucleotide polymorphisms (SNPs) of *HPSE* were shown to modulate mRNA and protein levels, possibly predicting disease outcomes.

**Methods and results:**

Given the potential role of heparanase in OC, the implications of three SNPs - rs11099592, rs4364254 and rs4693608 - were investigated in OC patients. In the discovery cohort, rs11099592 TT genotype and rs4364254 C allele carriers showed lower survival time than their counterparts (log-rank test, *p* = 0.025 and *p* = 0.001, respectively). Validation cohort analysis confirmed the worse prognosis associated with the rs11099592 T allele and the rs4364254 C allele in non-serous (log-rank test, *p* = 0.016) and platinum-resistant (log-rank test, *p* = 0.044) OC patients, respectively. The rs4364254 C allele was associated with reduced *HPSE* expression in peripheral blood components (χ^2^ test, *p* = 0.005), suggesting a protective role for HPSE in OC patients.

**Conclusions:**

*HPSE* rs11099592 and rs4364254 showed prognostic value, with T and C allele carriers, respectively, displaying worse clinical outcomes. These results indicate that heparanase could enable a tumour microenvironment shift towards a less aggressive cancer behaviour, facilitating leukocyte migration and anti-tumour responses. Further research should explore the dual mechanisms of this protein to improve OC management.

## Introduction

Heparanase, encoded by the homonymous gene located at 4q21.23, is the only enzyme in mammals capable of degrading heparan sulfate (HS), which is the primary component of the outer layer of the plasma membrane that covers endothelial cells, as well as a crucial constituent of the extracellular matrix (ECM) [1, 2]. Beyond its basic structure, HS can exist in the form of a proteoglycan (HSPG) when multiple HS chains are covalently attached to a protein core [3]. Additionally, other glycosaminoglycans (GAGs) that form proteoglycans, such as hyaluronic acid, and keratan and chondroitin sulphates, along with collagens, laminin, elastin and fibronectin, also make up the ECM [4]. HS plays a critical role in the integrity and organisation of the ECM, acting as a bridging link between its components [3, 5]. By cleaving HS, heparanase facilitates the remodelling of the subendothelial basal membrane and the ECM, an essential initial step in the formation of new vessels from pre-existing ones, consequently promoting endothelial cell migration and sprouting [2, 3, 6]. Furthermore, the heterogeneous structure characteristic of HS enables it to interact with various growth factors and other proteins, regulating not only their storage but also their access, function, and mode of action [5]. This broad spectrum of possible interactions accounts for the wide range of signalling pathways activated upon the release of these molecules through HPSE degradation, such as cell proliferation, tissue repair and angiogenesis [3, 5, 7].

Uncontrolled enzymatic activity of heparanase can support pathological processes, including infections and malignancy [8]. In the latter case, in addition to promoting the supply of nutrients and oxygen to tumour cells, heparanase can enhance cancer aggressiveness by inducing the expression of pro-angiogenic [vascular endothelial growth factors (VEGFs) A and C, and metalloproteinases 9 (MMP-9)], pro-inflammatory [tumour necrosis factor (TNFα) and interleukins 1 (IL-1) and 6 (IL-6)], and pro-coagulant factors (such as tissue factor) [2, 5]. *HPSE* is frequently overexpressed in oncological contexts, such as gastric, pancreatic, bladder, lung, and notably, ovarian carcinomas, corroborating its role in aggressiveness and disease recurrence [9]. In fact, the prognostic value of heparanase has already been reported for different cancer models, including its potential as a biomarker for evaluating surgery effects and survival outcomes in ovarian cancer (OC) [10, 11]. Furthermore, heparanase contributes to a pro-thrombotic environment, triggered by the release of tissue factor pathway inhibitor (TFPI) from the vessel wall, and subsequent induction of tissue factor (TF), further emphasising the intricacy of its connection with OC [12]. Indeed, among solid tumours, OC, like other gynaecological malignancies, is classified as a high-risk tumour for venous thromboembolism (VTE), according to cancer-associated thrombosis (CAT) scores such as the Khorana Score (KS) [13, 14].

Beyond classical actions, heparanase is increasingly recognised for its ability to modulate the tumour immune microenvironment by regulating the recruitment and activity of immune cells. This reflects the complex biology of heparanase, where its pro-malignant enzymatic activity is balanced by immune-associated functions that may inhibit tumour progression [11, 15, 16]. Therefore, while mainly considered a pro-tumourigenic and promising therapeutic target, heparanase’s potential anti-tumour effects warrant further investigation to fully capture its role in cancer biology and oncology treatments. In OC, where extensive peritoneal dissemination and immune evasion remain major therapeutic challenges, heparanase emerges as a particularly relevant molecule.

Due to late-stage diagnosis, OC stands as the most lethal malignancy among gynaecologic tumours on a global scale. The high rates of chemoresistance and tumour recurrence, combined with the high degree of heterogeneity of OC, further undermine current treatment strategies, with most countries reporting 5-year survival rates below 50% [17–23]. In the current landscape, exploring intricate mechanisms such as the interplay between heparanase, thrombosis and OC progression may hold the key to improving these dismal outcomes [19, 20]. Existing evidence demonstrates that genetic variations in *HPSE* modulate messenger RNA (mRNA) and protein levels, possibly serving as stable predictors of disease outcomes [24]. Given heparanase’s prominent role in ovarian tumourigenesis, further reinforced by its pro-thrombotic activity, these variations may affect survival and CAT susceptibility among OC patients. In this context, the present study investigated the role of three relevant *HPSE* single-nucleotide polymorphisms (SNPs) in OC patients, aiming to advance personalised and more effective disease management.

## Materials and methods

### Discovery and validation cohorts

A retrospective hospital-based cohort study was carried out at the Department of Gynaecology and Oncology of the Portuguese Oncology Institute of Porto (IPO Porto), enrolling histologically diagnosed epithelial OC (EOC) patients of European ancestry with admittances for first-line treatment from March 2017 to December 2023. Patients under the age of 18, seeking only a second opinion, or with follow-up elsewhere, were excluded, and a final cohort of 98 EOC patients (cohort A) with available biological material was established. Upon acceptance of participation, a written consent was handed to and signed by each patient according to the principles of the Helsinki Declaration. This study received approval from the ethics committee at IPO Porto (CES IPO: 69/021). The staging of all EOC cases was performed according to the International Federation of Gynaecology and Obstetrics (FIGO) Cancer Report 2021 [25]. Additionally, tumour response to chemotherapy was assessed using the Response Evaluation Criteria in Solid Tumours (RECIST) version 1.1 [26]. Demographic, clinicopathological, and follow-up data were obtained by reviewing the medical records of all patients. The study had a median follow-up of 25.5 months.

In cohort A, the average age of the participants was 63.2 years (range, 26–88 years), with the majority being post-menopausal (80.6%, *N* = 79) and diagnosed at FIGO stages III and IV (75.5%, *N* = 74). Most tumours were serous in type (83.7%, *N* = 82) and 41.8% of the patients (*N* = 41) underwent standard treatment - cytoreductive surgery followed by carboplatin/cisplatin-based chemotherapy with paclitaxel. Complete/optimal surgical resection was achieved in 45 patients (45.9%). CAT was characterised as a VTE event occurring between six months before and two years after the OC diagnosis [22, 27]. Among the 98 patients, information concerning CAT was available only for 80 of them, with 17 (21.3%) presenting the disease. Regarding thrombosis-related features, patients were evaluated for multiple non-mutually exclusive parameters. Excluding missing values for each test, 40 patients (46.0%) had a high-risk KS (KS ≥ 2); 38 (49.4%) presented high activated partial thromboplastin time (aPTT; ≥27.1 s); 41 (50.6%) high prothrombin time (PT; ≥14.2 s); and 39 (48.8%) high international normalised ratio (INR; ≥1.1).

Validation for SNPs’ analysis was conducted with an independent retrospective cohort – cohort B. This cohort included patients admitted at the same institution for first-line treatment from January 1996 to December 2012. Inclusion and exclusion criteria, disease staging and evaluation protocols for cohort B were consistent with those used for cohort A. A total of 331 EOC patients, for whom biological material was available, were enrolled. Like cohort A, each patient provided written consent following the principles of the Helsinki Declaration before their recruitment. This study received approval from the ethics committee at IPO Porto (CES IPO:286/2014). The mean follow-up in this cohort was 49.4 months.

In cohort B, the mean age of the enrolled patients was 55 years (range, 21–80 years). The following results were obtained considering the group of patients with valid information. Like cohort A, most of the patients were post-menopausal (64.6%, *N* = 203) and diagnosed at advanced cancer stages (FIGO stages III/IV; 61.3%, *N* = 196). Regarding the histological subtype, 56.7% (*N* = 187) were diagnosed with serous, 12.7% (*N* = 42) with clear cell, 10.3% (*N* = 34) with endometrioid, 9.7% (*N* = 32) with mucinous and the other 10.6% (*N* = 35) with less common tumour subtypes. Among the serous tumour types, 16.1% (*N* = 24) were low grade and 83.9% (*N* = 125) were high grade. Concerning therapeutic management, most patients underwent standard treatment (92.7%, *N* = 307), with cytoreductive surgery followed by chemotherapy with a combination of paclitaxel and carboplatin (*N* = 171) or cisplatin (*N* = 136). Neoadjuvant chemotherapy (*N* = 17, 5.1%), chemotherapy alone (*N* = 9, 2.7%) or only surgery (*N* = 4, 1.2%) were also considered as first-line treatment options. Complete or optimal surgical resection was achieved for 49.5% of the patients (*N* = 164). Regarding therapeutic response, most patients were highly platinum-sensitive (69.4%, *N* = 229), 9.7% (*N* = 32) were partially platinum-sensitive, 11.5% (*N* = 38) were platinum-resistant, and 4.8% (*N* = 16) were platinum-refractory. Notably, information on CAT events was not available for this cohort.

### Sample collection and nucleic acid extraction

Venous blood samples were collected in EDTA tubes before the initiation of the first line of chemotherapy. DNA was isolated using the QIAamp DNA Blood Mini Kit (Cat. No. 51106, Qiagen, Hilden, Germany), and RNA was extracted from peripheral blood components (PBCs) using the GRS RNA kit - Blood & cultured cells (#GK08.0100, Grisp Research Resolutions^®^, Porto, Portugal), following manufacturers’ protocols. Nucleic acid concentration and sample purity were confirmed using a NanoDrop spectrophotometer (Thermo Fisher Scientific, Waltham, MA, USA). DNA and RNA were stored at − 20 °C and − 80 °C, respectively, until further analysis.

### Polymorphism selection and genotyping

Among tumour biomarkers’ range, SNPs, alongside other genetic polymorphisms, offer the advantage of being stable DNA features remaining unaffected by cancer characteristics and treatment conditions [28]. Due to their clinical value and feasible applicability, three *HPSE* SNPs were chosen to be evaluated in this study: rs4364254, rs4693608 and rs11099592 (Table 1). The SNPs were selected based on their impact on heparanase expression or activity, relevance to cancer and/or cardiovascular diseases, availability of TaqMan^®^ SNP genotyping assays and the minor allele frequency (MAF ≥ 10%). Variants in strong linkage disequilibrium (r²>90%) were excluded. The three selected SNPs are extensively studied in the literature and have been pinpointed to have roles in several malignancies [29].

Polymorphism genotyping was performed using the StepOne Plus qRT-PCR system (Applied Biosystems^®^) with TaqMan allelic discrimination technology. Each PCR reaction mix (6.0 µL) contained 2.5 µL of TaqPath™ ProAmp™ Master Mix (1×), 2.375 µL of sterile water, 0.125 µL of TaqMan^®^ SNP genotyping assay, and 1.0 µL of genomic DNA. Thermal cycling conditions were as follows: 10 min at 95 °C for polymerase activation, 15 s at 95 °C for DNA denaturation (45 cycles), and 1 min at 60 °C for primer pairing and extension. Measures of quality control were carried out as described elsewhere [30].

### cDNA conversion and gene relative quantification

**Fig. 1:**
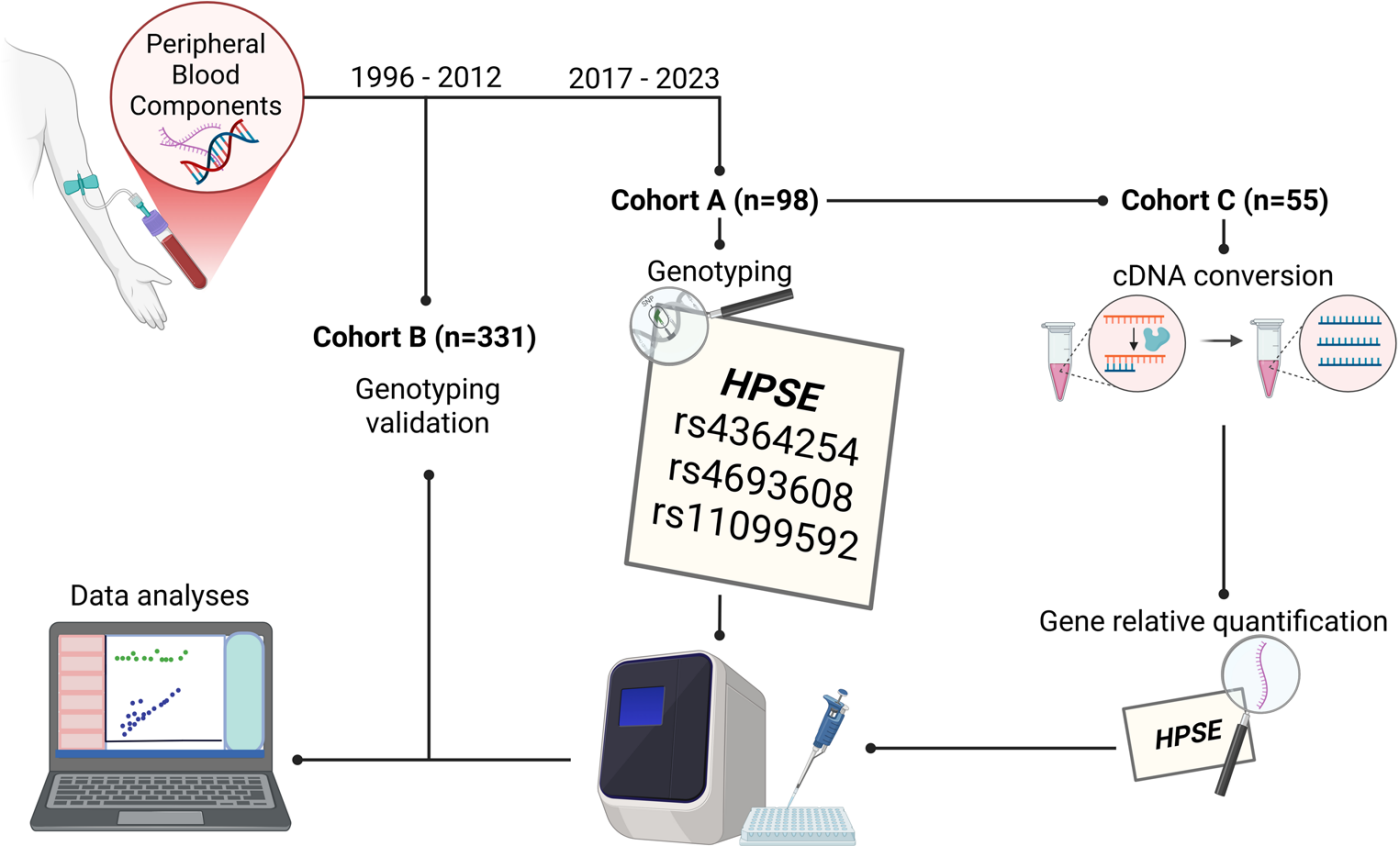
Methodology scheme depicting the three cohorts used in the present study

The HPSE expression was conducted to assess the impact of the evaluated SNPs on gene expression levels. This analysis was performed in a subsampled cohort A, comprised of 55 EOC patients, deemed cohort C. The exclusion criteria applied to cohort A were patients who: (1) had a history of malignancies before or after OC diagnosis; (2) were breastfeeding or pregnant at the time of diagnosis; (3) had a history of autoimmune diseases or were undergoing immunosuppressive therapies; (4) had acute infections at cancer diagnosis; (5) were undergoing anticoagulant treatment for diseases other than VTE; and (6) possessed the polymorphisms Factor V Leiden (F5 rs6025) and F2 (Factor II encoding gene) rs1799963. The rationale for the cohort selection and study design is illustrated in Fig. 1.

### Statistical analysis

Data analysis was performed using IBM SPSS Statistics software (version 29, IBM Corp., Armonk, NY, USA) for Windows. The Kolmogorov-Smirnov test was employed to assess data distribution. Continuous variables were categorised using the mean value as the cut-off for data with a normal distribution, or the median value for data with a non-normal distribution. The genotype frequencies of each SNP in this study were compared to those reported in the Iberian population (https://www.ensembl.org/index.html, accessed on 18th August 2024). The Hardy-Weinberg equilibrium (HWE) was assessed using the chi-square test (χ² test). 

Gene normalised-relative expression (-ΔCt) was calculated via the Livak method using GAPDH as the most suitable control. Severe outliers were removed based on the interquartile range (IQR). Four expression profiles were defined for analysis: A) low vs. high expression based on the median value (-ΔCt = −4.8); B) low, intermediate, and high expression defined by terciles (cut-offs: -ΔCt ≤ −5.3, −5.2 to −4.7, ≥ −4.6, respectively); C) low (first two terciles) vs. high (third tercile), and D) low (first tercile) vs. high (second and third terciles), as previously described.

The SNPs’ impact on the patient’s progression-free survival (PFS) and overall survival (OS) was assessed. PFS was calculated as the time from tumour diagnosis to recurrence, progression, death, or the last clinical evaluation, while OS referred to the period from diagnosis to death or last evaluation. Survival curves were generated using the Kaplan-Meier method, and the log-rank test was used to compare survival probabilities. The most appropriate genetic model (dominant or recessive) for each variant was selected based on survival curve analysis under the additive model. Cox proportional hazard models were employed to estimate the risks of tumour progression and patient death. Validation of the SNP analysis was conducted considering the entire cohort B and subgroups based on patients’ age; hormonal status (pre- or post-menopause) at diagnosis; cancer stage; histological subtype; OC differentiation grade; surgical resection, and platinum sensitivity. 

Statistical tests were two-sided with a 5% significance level, and p-values between 0.050 and 0.060 were considered marginally significant.

### cDNA conversion and gene relative quantification

The HPSE expression was conducted to assess the impact of the evaluated SNPs on gene expression levels. This analysis was performed in a subsampled cohort A, comprised of 55 EOC patients, deemed cohort C. The exclusion criteria applied to cohort A were patients who: 1) had a history of malignancies before or after OC diagnosis; 2) were breastfeeding or pregnant at the time of diagnosis; 3) had a history of autoimmune diseases or were undergoing immunosuppressive therapies; 4) had acute infections at cancer diagnosis; 5) were undergoing anticoagulant treatment for diseases other than VTE; and 6) possessed the polymorphisms Factor V Leiden (F5 rs6025) and F2 (Factor II encoding gene) rs1799963. The rationale for the cohort selection and study design is illustrated in Fig. 1.

Total RNA samples were utilised as templates to generate the complementary DNA (cDNA) strands, using the High-Capacity cDNA Reverse Transcription Kit (Applied Biosystems®, Carlsbad, CA, USA), as previously described [30].

Each reaction for gene expression analysis was executed with the StepOne Plus qRT-PCR system (Applied Biosystems®, Foster City, CA, USA) using a 10.0 µL mixture containing: 5.0 µL of 2× TaqManTM Gene Expression Master Mix and 0.5 µL of TaqManTM 20× Gene Expression Assay Hs00935036_m1, both by Applied Biosystems® (Foster City, CA, USA); 3.0 µL of nuclease-free water and 1.5 µL of cDNA sample. Adding to these, glyceraldehyde-3-phosphatedehydrogenase (GAPDH) and hypoxanthine phosphoribosyl transferase 1 (HPRT1) were evaluated as endogenous controls with the assays Hs03929097_g1 and Hs02800695_m1, respectively. Thermal cycling conditions included 50 °C for 2 min, 95 °C for 10 min, followed by 45 cycles of 95 °C for 15 s and 60 °C for 1 min. Negative controls were included, and all samples were run in triplicate. Measures of quality control were carried out as described elsewhere [30, 31]. The Thermo Fisher Connect platform (Thermo Fisher Scientific, Waltham, MA, USA) was employed for data analysis.

### Statistical analysis

Data analysis was performed using IBM SPSS Statistics software (version 29, IBM Corp., Armonk, NY, USA) for Windows. The Kolmogorov-Smirnov test was employed to assess data distribution. Continuous variables were categorised using the mean value as the cut-off for data with a normal distribution, or the median value for data with a non-normal distribution. The genotype frequencies of each SNP in this study were compared to those reported in the Iberian population (https://www.ensembl.org/index.html, accessed on 18th August 2024). The Hardy-Weinberg equilibrium (HWE) was assessed using the chi-square test (χ² test). 

Gene normalised-relative expression (-ΔCt) was calculated via the Livak method using GAPDH as the most suitable control. Severe outliers were removed based on the interquartile range (IQR). Four expression profiles were defined for analysis: A) low vs. high expression based on the median value (-ΔCt = −4.8); B) low, intermediate, and high expression defined by terciles (cut-offs: -ΔCt ≤ −5.3, −5.2 to −4.7, ≥ −4.6, respectively); C) low (first two terciles) vs. high (third tercile), and D) low (first tercile) vs. high (second and third terciles), as previously described.

The SNPs’ impact on the patient’s progression-free survival (PFS) and overall survival (OS) was assessed. PFS was calculated as the time from tumour diagnosis to recurrence, progression, death, or the last clinical evaluation, while OS referred to the period from diagnosis to death or last evaluation. Survival curves were generated using the Kaplan-Meier method, and the log-rank test was used to compare survival probabilities. The most appropriate genetic model (dominant or recessive) for each variant was selected based on survival curve analysis under the additive model. Cox proportional hazard models were employed to estimate the risks of tumour progression and patient death. Validation of the SNP analysis was conducted considering the entire cohort B and subgroups based on patients’ age; hormonal status (pre- or post-menopause) at diagnosis; cancer stage; histological subtype; OC differentiation grade; surgical resection, and platinum sensitivity. 

Statistical tests were two-sided with a 5% significance level, and p-values between 0.050 and 0.060 were considered marginally significant. 

**Table 1:** Selected *HPSE* SNPs and the respective TaqMan^®^ Genotyping Assays

SNP	Functional consequence	MAF in Iberians * (MA)	TaqMan® SNP Genotyping Assay
rs4364254 (C/T)	Intronic	32.2% (C)	C___8416664_10
rs4693608 (G/A)	Intronic	46.7% (A)	C__30667102_10
rs11099592 (T/C)	Missense	29.9% (T)	C__31870510_10

*Abbreviations*: MA, minor allele; MAF, minor allele frequency; SNP, single-nucleotide polymorphism. * According to the Ensembl database (last accessed on the 18th of August of 2024).

## Results

### Distribution of SNP genotypes

The distribution of the variants’ genotypes is represented in Table 2. Notably, all the SNPs were in HWE (χ^2^ test, *p* > 0.050), demonstrating no significant deviation from expected genotype frequencies.

### HPSE SNPs and gene expression

In cohort C, rs4364254 demonstrated a significant association with HPSE expression in PBCs. In the additive model, based on expression profile C (with the third tercile regarded as a high expression), the C allele was associated with a significantly lower HPSE expression than the TT genotype (CC vs. CT vs. TT; x² test, p = 0.018). Under the dominant model, the presence of the C allele was similarly linked to significantly reduced HPSE expression, both considering the profile B (low vs. intermediate vs. high) (CC/CT vs. TT; x² test, p = 0.014) and profile C (CC/CT vs. TT; x² test, p = 0.005). Overall, the HPSE rs4364254 C allele was consistently associated with reduced HPSE gene expression in PBCs. As for the remaining SNPs, no significant association with the gene expression levels was found, nor was any discernible trend observed (Table 3).

### HPSE SNPs and clinical features of OC patients

In the discovery cohort (cohort A), the SNPs rs4364254 and rs11099592 were associated with various demographic and clinicopathological features. The rs4364254 C allele was significantly associated with older patient age at OC diagnosis in both the additive (x² test, p = 0.049) and dominant (CC/CT vs. TT; x² test, p = 0.047) models. Additionally, the C allele was more common in patients with a high revised KS (KS ≥ 2; x² test, p = 0.034). A marginal association was also observed with aPTT, suggesting that the C allele is more likely linked to lower aPTT, while the TT genotype is associated with a prolonged assay (CC/CT vs. TT; x² test, p = 0.053). However, no SNP showed an association with VTE susceptibility. Regarding rs11099592, it was marginally associated with a history of other tumours in the additive model (x² test, p = 0.053), being more prevalent in patients without such a history. Like the genetic variants, KS showed a poor predictive value for VTE events, regardless of the cut-off considered (x² test, p ≥ 0.050).

### HPSE SNPs and OC patient outcomes

**Fig. 2:**
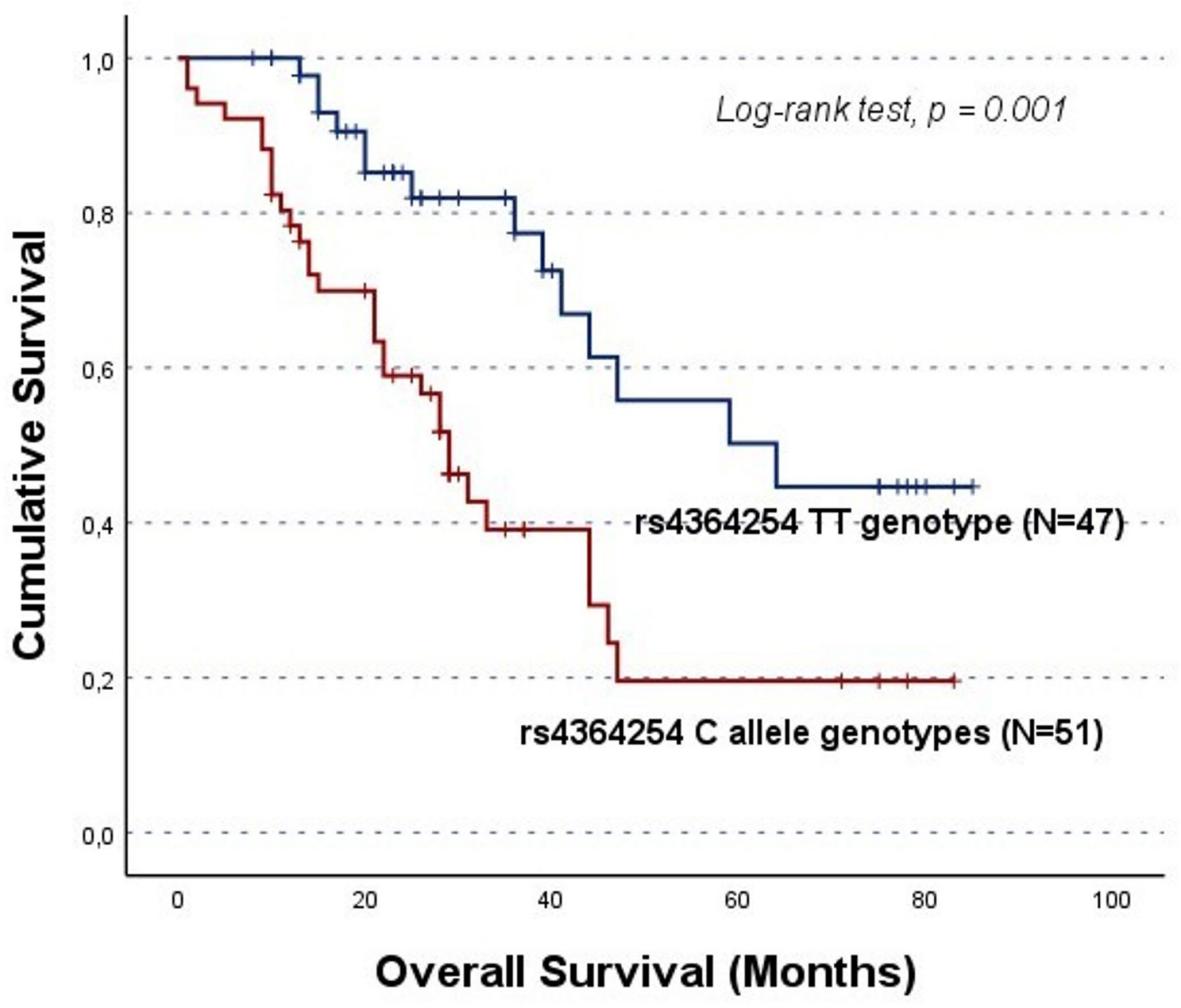
Overall survival (OS) by Kaplan-Meier and log-rank test for OC patients in cohort A, according to *HPSE* rs4364254 genotype distribution. The C allele carriers showed lower OS compared to TT genotype carriers (CC/CT vs. TT; log-rank test, *p* = 0.001). The mean OS for C allele carriers was 36.1 ± 4.5 months, while for TT genotype carriers was 59.0 ± 5.2 months

**Fig. 3:**
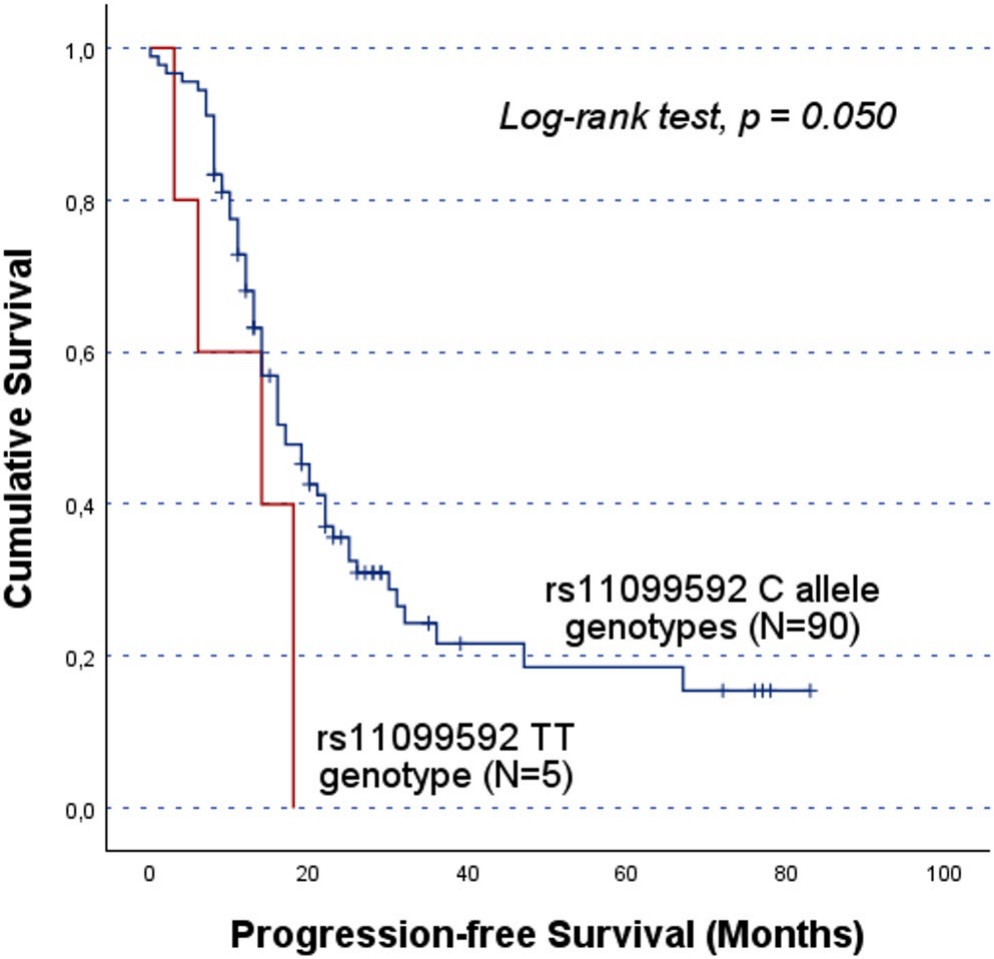

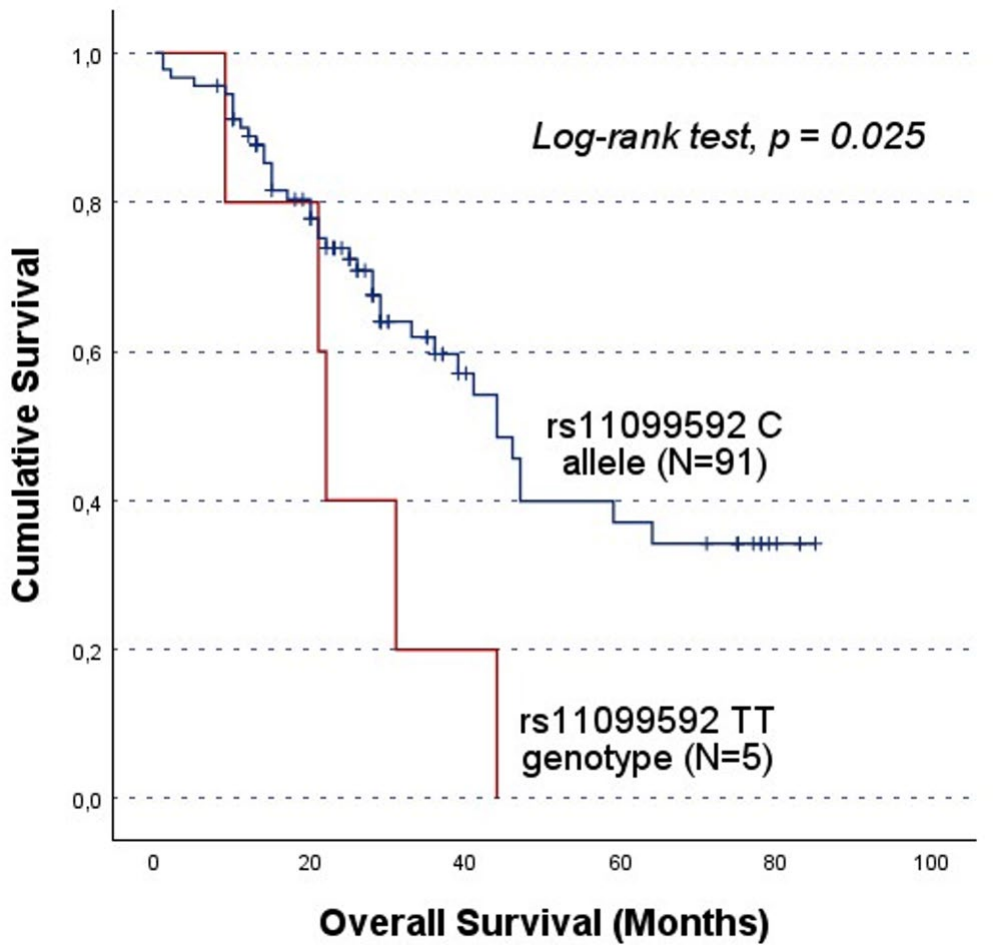
Progression-free survival (PFS) and overall survival (OS) by Kaplan-Meier and log-rank test for OC patients in cohort A, according to *HPSE* rs11099592 genotype distribution. The TT genotype group showed lower survival times compared to C allele carriers. **A**) The mean PFS for C allele carriers was 28.8 ± 3.3 months, while TT carriers presented a mean PFS of 11.8 ± 3.1 months (CC/CT vs. TT; log-rank test, *p* = 0.050). **B**) The mean OS for C allele carriers was 48.9 ± 3.9 months, while for TT genotype carriers the mean OS was 25.4 ± 5.8 months (CC/CT vs. TT; log-rank test, *p* = 0.025). However, both findings should be evaluated carefully, given the underrepresentation of the TT genotype The significant results concerning the impact of the SNPs on patients’ prognosis in cohort A were validated in the independent cohort B. While no significant association was detected in the entire cohort B, stratified analyses confirmed the association between the rs4364254 C and rs11099592 T alleles and poorer clinical outcomes. Among the patients resistant to platinum, the rs4364254 C allele was associated with lower OS compared to the TT genotypes (CC/CT vs.TT; mean OS of 19.9 ± 2.4 months and 28.8 ± 3.4 months; log-rank test, *p* = 0.044; Fig. 4). As for rs11099592, non-serous OC patients carrying the T allele presented a worse OS than their counterparts with the CC genotype (TT/CT vs. CC; mean OS of 45.1 ± 3.3 months and 53.5 ± 1.6 months, respectively; log-rank test, *p* = 0.016; Fig. 5)

In cohort A, the rs4364254 C allele carriers showed lower OS compared to the TT genotype group (CC/CT vs. TT; 36.1 ± 4.5 months and 59.0 ± 5.2 months, respectively, log-rank test, p = 0.001; Fig. 2). Moreover, rs11099592 was found to be associated with both PFS and OS. Specifically, TT genotype carriers had a lower PFS than their counterparts (CC/CT vs. TT; mean PFS of 28.8 ± 3.3 months and 11.8 ± 3.1 months, log-rank test, p = 0.050; Fig. 3 A). Likewise, TT genotype carriers showed lower OS (CC/CT vs. TT; mean OS of 25.4 ± 5.8 months and 48.9 ± 3.9 months, respectively, log-rank test, p = 0.025; Fig. 3B). However, it should be noted that these survival analyses are based on a limited number of TT genotype carriers (N = 5).

**Fig. 4:**
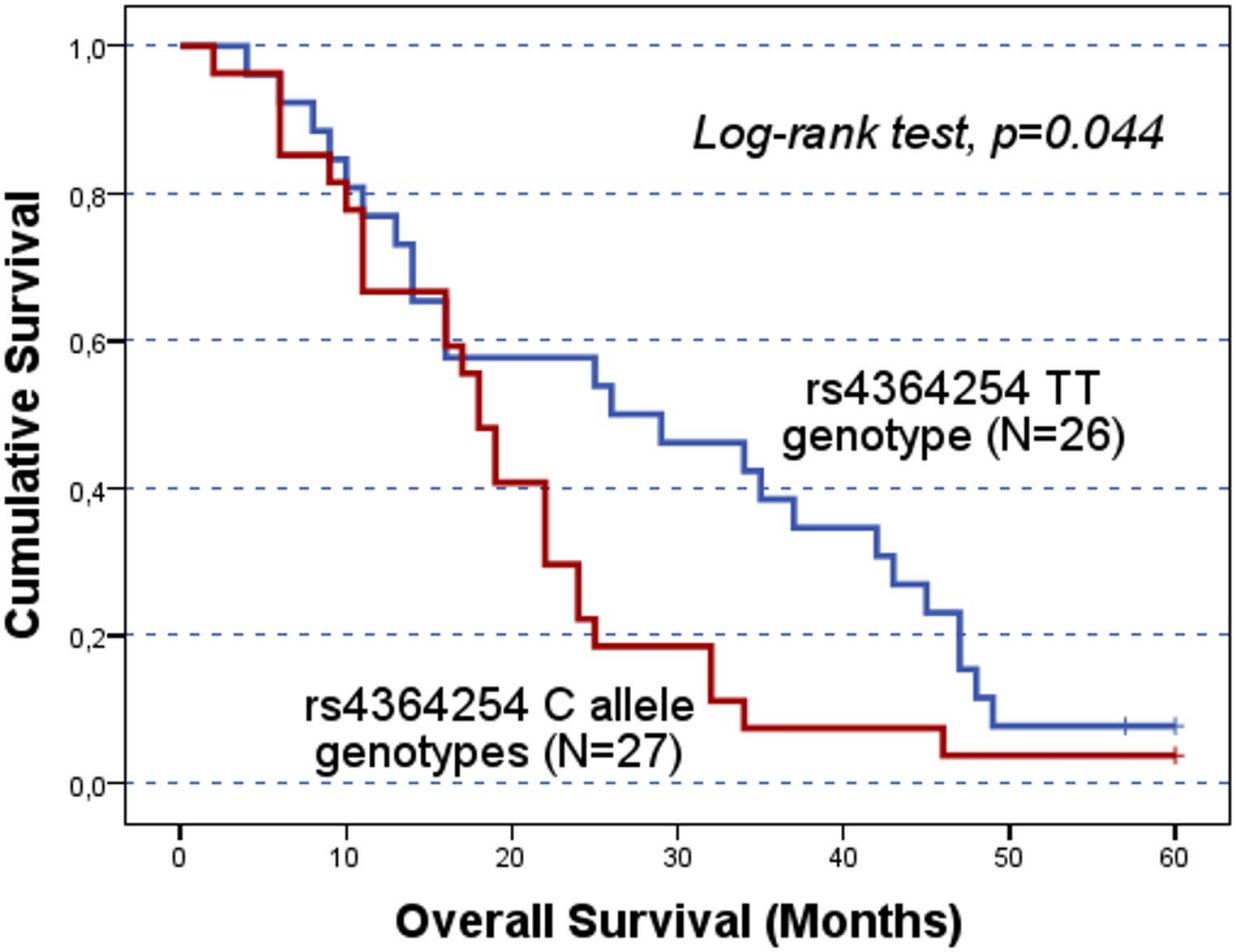
Overall survival (OS) by Kaplan-Meier and log-rank test for cohort B platinum-resistant OC patients, according to *HPSE* rs4364254 genotype distribution. C allele carriers had a worse OS than TT allele carriers (CC/CT vs.TT; log-rank test, *p =* 0.044). The mean OS for C allele and TT genotype carriers was 19.9 ± 2.4 months and 28.8 ± 3.4 months, respectively

**Fig. 5:**
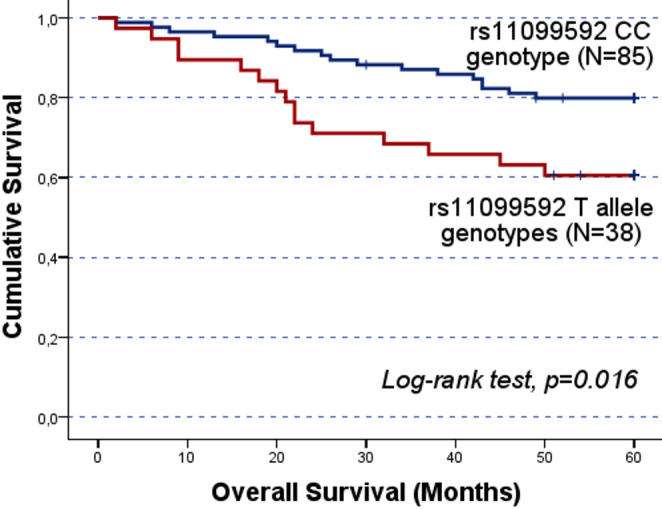
Overall survival (OS) by Kaplan-Meier and log-rank test for cohort B non-serous OC patients, according to genotype distribution for *HPSE* rs11099592. T allele carriers presented a worse OS than CC allele carriers (TT/CT vs. CC; log-rank test, *p =* 0.016). T allele and CC genotype carriers had a mean OS of 45.1 ± 3.3 months and 53.5 ± 1.6 months, respectively

The significant results concerning the impact of the SNPs on patients’ prognosis in cohort A were validated in the independent cohort B. While no significant association was detected in the entire cohort B, stratified analyses confirmed the association between the rs4364254 C and rs11099592 T alleles and poorer clinical outcomes. Among the patients resistant to platinum, the rs4364254 C allele was associated with lower OS compared to the TT genotypes (CC/CT vs.TT; mean OS of 19.9 ± 2.4 months and 28.8 ± 3.4 months; log-rank test, p = 0.044; Fig. 4). As for rs11099592, non-serous OC patients carrying the T allele presented a worse OS than their counterparts with the CC genotype (TT/CT vs. CC; mean OS of 45.1 ± 3.3 months and 53.5 ± 1.6 months, respectively; log-rank test, p = 0.016; Fig. 5).

## Discussion

Long-term survival rates for OC remain poor, despite progress in treatment options, highlighting the persistent challenges in effectively managing this disease. Late-stage diagnoses, therapy resistance and high recurrence rates continue to hinder meaningful advancements, emphasising the urgent need to rethink strategies and explore new avenues, such as the identification of robust prognostic biomarkers [19, 20, 22, 32, 33]. In this context, the interplay between heparanase, VTE, and OC could be the key to these needed advances, facilitating more personalised treatment approaches. Heparanase plays a pivotal role in cancer progression, influencing multiple hallmarks of the disease, particularly in OC, where it contributes to angiogenesis and tumour invasion. At the same time, the endothelial permeability promoted by heparanase fosters VTE, a major complication in cancer patients [34, 35]. For OC, this is reflected in high CAT scores, largely influenced by the surgery location and type of treatment involved. Building on this, the present study focused on investigating the role of *HPSE* SNPs in OC prognosis, aiming to further advance personalised and more effective disease management.

To begin with, the variant *HPSE* rs4364254, located in intron 9, involves the substitution of a cytosine (C) with a thymine (T) at nucleotide position 8,718,418 [36, 37]. The T allele has been associated with higher *HPSE* expression levels, which was confirmed in the present study (CC/CT vs. TT; χ² test, *p =* 0.005) [38]. Notably, among the three *HPSE* SNPs evaluated (rs4364254, rs4693608 and rs11099592), rs4364254 was the only one to show a significant association with *HPSE* expression in PBCs. Although the underlying mechanism remains unclear, existing evidence suggests that *HPSE* rs4364254 is located within an insulator region of DNA, and thus it might alter the function of this regulatory element, affecting gene expression [39]. The combination of *HPSE* rs4364254 with rs4693608 can modulate the gene transcriptional activity. The latter is an intronic variant located in an enhancer, involving a guanine (G) to adenine (A) substitution, which has been reported to affect gene expression [37]. Based on haplotype analyses registered in the literature, when individual alleles are considered, the A allele of rs4693608 is linked to an increased *HPSE* expression [36]. However, as previously mentioned, this association was not observed in our study.

Intriguingly, in a previous study also assessing *HPSE* SNPs and gene expression in PBCs, lower *HPSE* expression was associated with higher plasmatic heparanase (pHPSE), and vice versa, in healthy individuals. This unexpected dynamic may be explained by the complex trafficking, processing, and secretion of the protein. *HPSE* mRNA leads to the synthesis of the protein pro-heparanase, which then undergoes rapid processing and activation in the Golgi and lysosomes, followed by secretion – pHPSE. In a case of high *HPSE* expression, much of the active pHPSE binds to the ECM or cell surface, limiting its detection in plasma. Thus, while mRNA levels may be higher, pHPSE detection reflects only the active, secreted form, detached from the ECM or cell surface. Additionally, high pHPSE levels may lead to a reduction in mRNA expression through a feedback mechanism. Assuming this reported negative correlation applies to other *HPSE* SNPs, this data may explain the obtained results regarding *HPSE* mRNA, genotype distributions and further associations in the present study [24].

Regarding the association with patient characteristics, the rs4364254 C allele - linked to lower *HPSE* levels - was associated with a single coagulation-related clinical parameter (high-risk KS ≥ 2; χ² test, *p =* 0.034). Although this may suggest that the rs4364254 C allele unexpectedly reflects a pro-thrombotic profile, it is worth noting that neither the SNP nor KS showed a significant association with VTE [40]. Notably, there was a high prevalence of the rs4364254 C allele among patients diagnosed at advanced age (≥ 55 years) (CC/CT vs. TT; χ² test, *p =* 0.047). This age-related prevalence may partially explain the negative prognostic impact of the C allele, as disclosed further in the next paragraph.

Concerning OC patients’ prognosis, *HPSE* rs4364254 had a significant impact. Namely, compared to TT genotype carriers, patients with the C allele - associated with lower *HPSE* expression - exhibited lower OS in cohort A and stratified cohort B (log-rank test, *p* = 0.001 and *p* = 0.044, respectively). This negative impact of the rs4364254 C allele is not consistent with previous cancer studies with endometrial and gastric cancer patients, where TT genotypes - linked to higher *HPSE* expression - were associated with cervical invasion and poorer survival, respectively [37, 41]. However, this effect aligns with the negative correlation between *HPSE* expression and pHPSE levels reported in the literature [24, 36]. The reduced OS observed among C allele carriers compared to TT genotype carriers in the validation study, specifically among platinum-resistant patients (log-rank test, *p* = 0.044), suggests that molecular pathways driven by lower *HPSE* expression, potentially coupled with elevated pHPSE levels, may contribute to a tumour microenvironment (TME) that is more resilient to the cytotoxic effects of platinum-based therapies. From a clinical standpoint, this result highlights the potential of rs4364254 as a predictive biomarker to stratify patient outcomes. Identifying C allele carriers among platinum-resistant patients could help anticipate a more aggressive disease course, underscoring the need for alternative non-platinum-based therapeutic strategies earlier in the clinical management.

Collectively, local action of heparanase is widely recognised for its pro-tumorigenic role, promoting angiogenesis, tumour invasion, and metastasis through the degradation of HS in the ECM. On one hand, *HPSE* expression by TME components, such as activated ECs, is deemed an aggressive phenotype marker, leading to poorer outcomes [5, 42, 43]. On the other hand, considering the systematic action of heparanase, the gene expression by leukocytes can paradoxically exhibit anti-tumourigenic effects under certain conditions. The cleavage of HS by heparanase is multifunctional, releasing growth factors and cytokines that, depending on the microenvironment, can enhance immune surveillance, facilitating tumour progression inhibition. Mayfosh et al. (2019) demonstrate that heparanase overexpression in T cells and NK cells enhances their migration and infiltration into tumours [44]. Whether gene and protein expression are indeed inversely correlated, or if heparanase, contrary to its well-established pro-tumourigenic role, could be exerting beneficial immunomodulatory effects, needs to be clarified. Considering this anti-tumourigenic role, lower *HPSE* associated with the rs4364254 C allele might limit this release, potentially impairing the body’s ability to mount an effective defence against the tumour. Thus, heparanase may play a role in shifting OC’s cold immune microenvironment to an immune-inflamed (hot) tumour phenotype [45]. Overall, additional studies are required to dissect the implications of the *HPSE* rs4364254 C allele in the clinical outcomes of OC patients. As for *HPSE* rs4693608, no prognostic value was detected.

Regarding rs11099592, this missense variation, located at intron 7, involves the substitution of a thymine (T) with a cytosine (C) [38]. This SNP significantly impacted OC patients’ prognosis, with C allele carriers presenting a higher PFS and OS compared to patients carrying the TT genotype in cohort A (log-rank test, *p* = 0.050 and *p* = 0.025, respectively). While the very small number of TT genotype carriers (*N* = 5) in this initial cohort requires these specific survival outcomes to be interpreted with caution, the biological relevance of the T allele was corroborated by our validation analyses. Among non-serous OC patients, the T allele showed a persistent association with a worse prognosis (log-rank test, *p* = 0.016). Much like the prognostic value observed for rs4364254 in platinum-resistant cases, by identifying non-serous OC patients carrying the T allele, clinicians could pinpoint a high-risk population that might benefit from closer surveillance and tailored therapeutic interventions.

Notably, the predominant impact on OS suggests that these presently studied specific genetic variations do not primarily dictate the immediate response to first-line treatments, but rather, they carry a cumulative biological weight throughout the disease trajectory. By modulating HPSE expression and its subsequent interaction with the TME, these variants likely drive long-term adaptive mechanisms, such as sustained immune evasion, long-term angiogenesis, or resistance to subsequent lines of therapy (as observed with rs4364254 in platinum-resistant patients).

Although no association between rs11099592 and *HPSE* expression was observed in this study, the literature suggests that the C allele enhances its levels [24, 36, 38]. This aligns with the pattern observed for rs4364254, where the allele linked to reduced expression correlates with a poorer prognosis. Although studies should clarify the distinct heparanase roles across different histological types of OC, this finding can be attributed to how *HPSE* interacts with the TME [10, 46]. Once more, these results not only challenge the conventional view of heparanase as exclusively pro-tumourigenic but also question the assumed inverse correlation between its gene and protein expression [24, 44, 47]. Interestingly, in a marginal association, the rs11099592 C allele was more prevalent in patients with a history of other tumours (χ² test, *p =* 0.053), aligning with the observation that elevated *HPSE* may facilitate tumourigenesis in different tissues through mechanisms like ECM remodelling and angiogenesis [29, 36]. Given these complex and contradictory roles, further investigation is crucial to determine how heparanase expression and function vary across tumour contexts and whether its modulation could offer therapeutic opportunities in OC. Further studies with larger and more diverse cohorts should incorporate the evaluation of immune infiltrate composition and pHPSE levels in parallel with *HPSE* expression, to dissect the complex dynamics between the genetic variants, transcriptional activity, protein levels, immune response and ovarian tumourigenesis. Notably, earlier research investigated both the mRNA and serum heparanase levels in OC patients and their effects on clinical characteristics. However, the interplay between *HPSE* expression and circulating protein levels was not clarified, and HPSE (both gene and protein expression levels) was proposed as a diagnostic biomarker for ovarian tumours [10]. Considering the differences between serum and plasma, the results from these studies underscore the complexity of heparanase. Additionally, exploring the gene expression across different cellular compartments, beyond PBCs, could provide further clarification into its specific sources and functional implications.

## Conclusion

In the cancer research field, heparanase has garnered significant interest due to its role in angiogenesis, tumour invasion, immune modulation and metastasis. Given the reported dual role of this enzyme in VTE and cancer progression, we aimed to assess the impact of *HPSE* SNPs on OC progression and patient survival. This study demonstrates that these polymorphisms, particularly rs4364254 and rs11099592, significantly impact OC patient survival. Challenging the conventional view of heparanase as solely pro-tumourigenic, our findings reveal that alleles linked to lower *HPSE* expression unexpectedly associate with worse clinical outcomes. While only the association between the rs4364254 C allele and reduced *HPSE* levels was confirmed in our cohort, the literature supports a similar effect for the rs11099592 C allele. Importantly, these adverse prognostic effects were validated in challenging subgroups, such as platinum-resistant and non-serous OC patients. These findings open new perspectives on the role of heparanase in OC progression and highlight the need for further studies to elucidate the interplay between its gene and protein expression and immune infiltration. Notably, recognising the complex, context-dependent functions of heparanase will be crucial for the future development of targeted therapies and the improvement of long-term survival in OC. Ultimately, integrating HPSE variants into standard molecular diagnostic panels could provide a dual-layered prognostic tool, enhancing the precision of personalised medicine in OC management.

**Table 2:** Genotype distribution of the *HPSE* SNPs in cohort A (*N* = 98)

SNP	MAFi * (MA)	MAFs (MA)	Genotype	*N* (%)	*N* total (%)
rs4364254	32.2% (C)	29.1% (C)	CC	6 (6.1)	98 (100)
			CT	45 (45.9)	
			TT	47 (48.0)	
rs4693608	46.7% (A)	48.5% (G)	GG	22 (22.4)	98 (100)
			AG	51 (52.0)	
			AA	25 (25.5)	
rs11099592	29.9% (T)	23.9% (T)	CC	55 (57.3)	96 (98)
			CT	36 (37.5)	
			TT	5 (5.2)	

*According to the Ensembl database (last accessed on 18th of August of 2024). Abbreviations: MA, minor allele; MAFi, minor allele frequency in the Iberian population; MAFs, minor allele frequency in the study cohort; SNP, single-nucleotide polymorphism

**Table 3:** Genotype distribution of each *HPSE* SNP (additive model) in cohort C (*N* = 55), according to the expression profile A of the respective gene

SNP	Genotype	Low expression** *N* (%)	High expression** *N* (%)
rs4364254 *	CC	3 (5.5)	1 (1.8)
	CT	24 (43.6)	5 (9.1)
	TT	10(18.2)	12 (21.8)
rs4693608	GG	6 (10.9)	7 (12.7)
	AG	13 (23.6)	14 (25.4)
	AA	8 (14.5)	9 (16.4)
rs11099592	CC	15(27.3)	15 (27.3)
	CT	12 (21.8)	9 (16.4)
	TT	0 (0.0)	4 (7.3)

* Statistically significant results for *HPSE* rs4364254 were found considering the expression profile C (χ², *p =* 0.018). ** *HPSE* expression levels were categorised as “low” or “high” based on the median normalised-relative gene expression value (-ΔCt = −4.8)

## Data Availability

The data presented in this study is available on request from the corresponding author.
