# Correction: Heparanase (HPSE) genetic variants as prognostic indicators in ovarian cancer: evidence from discovery and validation cohorts

**DOI:** 10.1007/s11033-026-11953-1

**Published:** 2026-06-03

**Authors:** Inês Guerra de Melo, Valéria Tavares, Joana Savva-Bordalo, Mariana Rei, Joana Liz-Pimenta, Deolinda Pereira, Rui Medeiros

**Affiliations:** 1 Molecular Oncology and Viral Pathology Group, Department of Pathology and Laboratory Medicine/RISE - Associate Laboratory (Health Research Network), IPO Porto Research Centre (CI-IPOP), Portuguese Oncology Institute of Porto (IPO Porto), Porto Comprehensive Cancer Centre Raquel Seruca (Porto.CCC), Porto, 4200-072 Portugal; 2 Faculty of Medicine, University of Porto (FMUP), Porto, 4200-072 Portugal; 3 Research Department, Portuguese League Against Cancer (NRNorte), Porto, 4200-172 Portugal; 4 Instituto de Ciências Biomédicas Abel Salazar (ICBAS), University of Porto, Porto, 4050-313 Portugal; 5 School of Medicine and Biomedical Sciences (EMCB), Fernando Pessoa University, Gondomar, 4420-096 Portugal; 6 Department of Medical Oncology, Portuguese Oncology Institute of Porto (IPO Porto), Porto, 4200-072 Portugal; 7 Department of Gynaecology, Portuguese Oncology Institute of Porto (IPO Porto), Porto, 4200-072 Portugal; 8 Department of Medical Oncology, Centro Hospitalar de Trás-os-Montes e Alto Douro (CHTMAD), Vila Real, 5000-508 Portugal; 9 Faculty of Health Sciences, Fernando Pessoa University, Porto, 4200-150 Portugal

**Correction to: Molecular Biology Reports (2026) 53:713**

10.1007/s11033-026-11891-y

In the original publication, Figs. 1, 2, 3, 4 and 5 were inadvertently interchanged, during production process. While the figure legends remain correct, the images themselves were swapped. The figures with their appropriate legends are presented below.

**Fig. 1:**
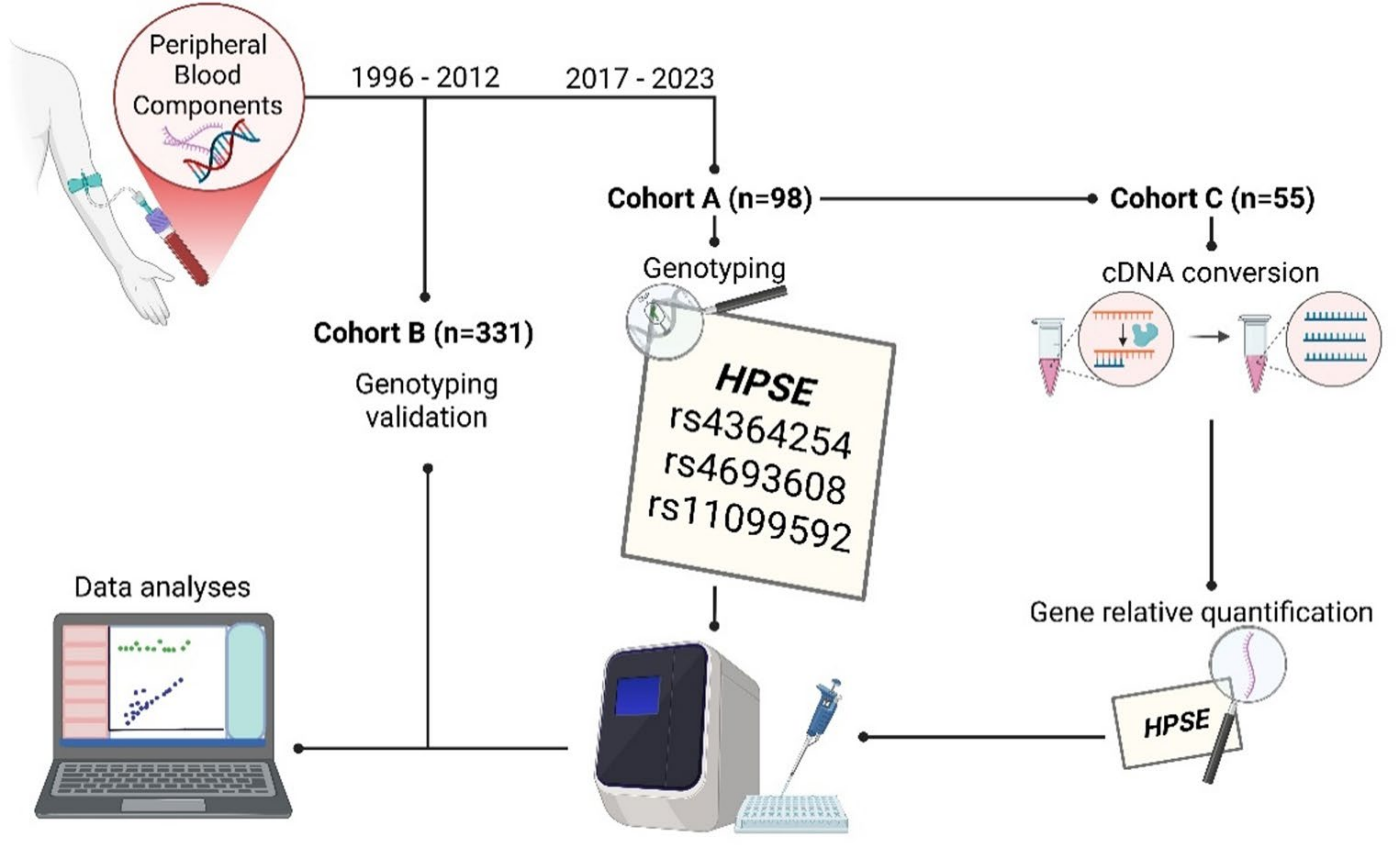
Methodology scheme depicting the three cohorts used in the present study

**Fig. 2:**
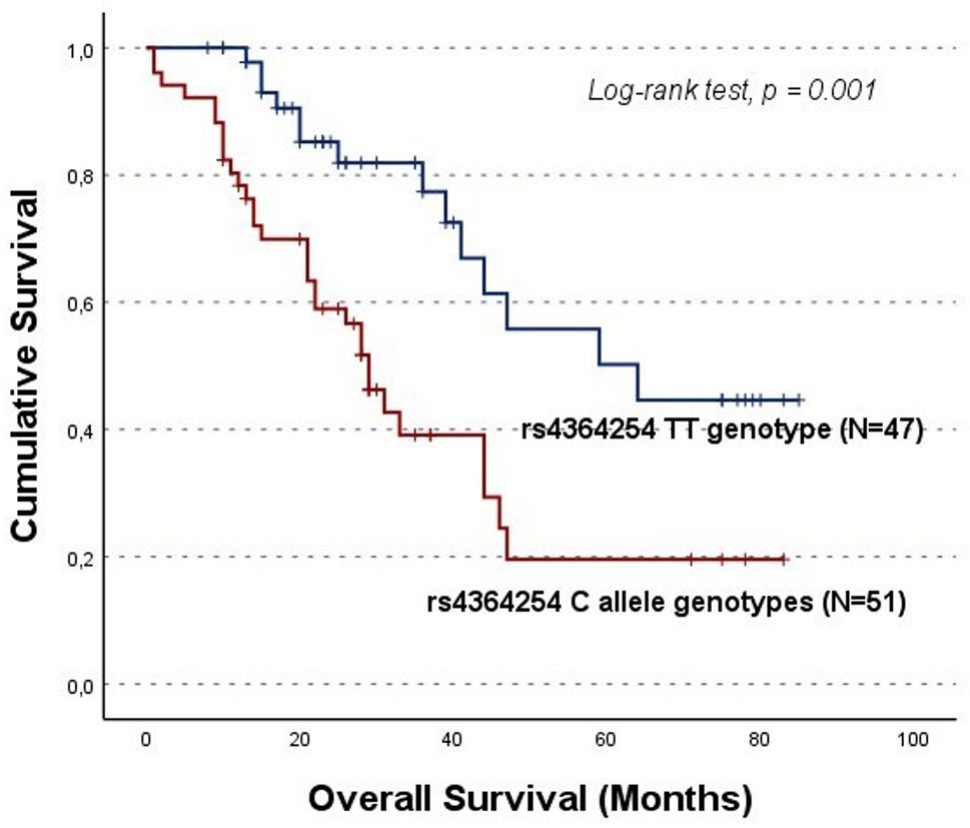
Overall survival (OS) by Kaplan-Meier and log-rank test for OC patients in cohort A, according to *HPSE* rs4364254 genotype distribution. The C allele carriers showed lower OS compared to TT genotype carriers (CC/CT vs. TT; log-rank test, *p* = 0.001). The mean OS for C allele carriers was 36.1 ± 4.5 months, while for TT genotype carriers was 59.0 ± 5.2 months

**Fig. 3:**
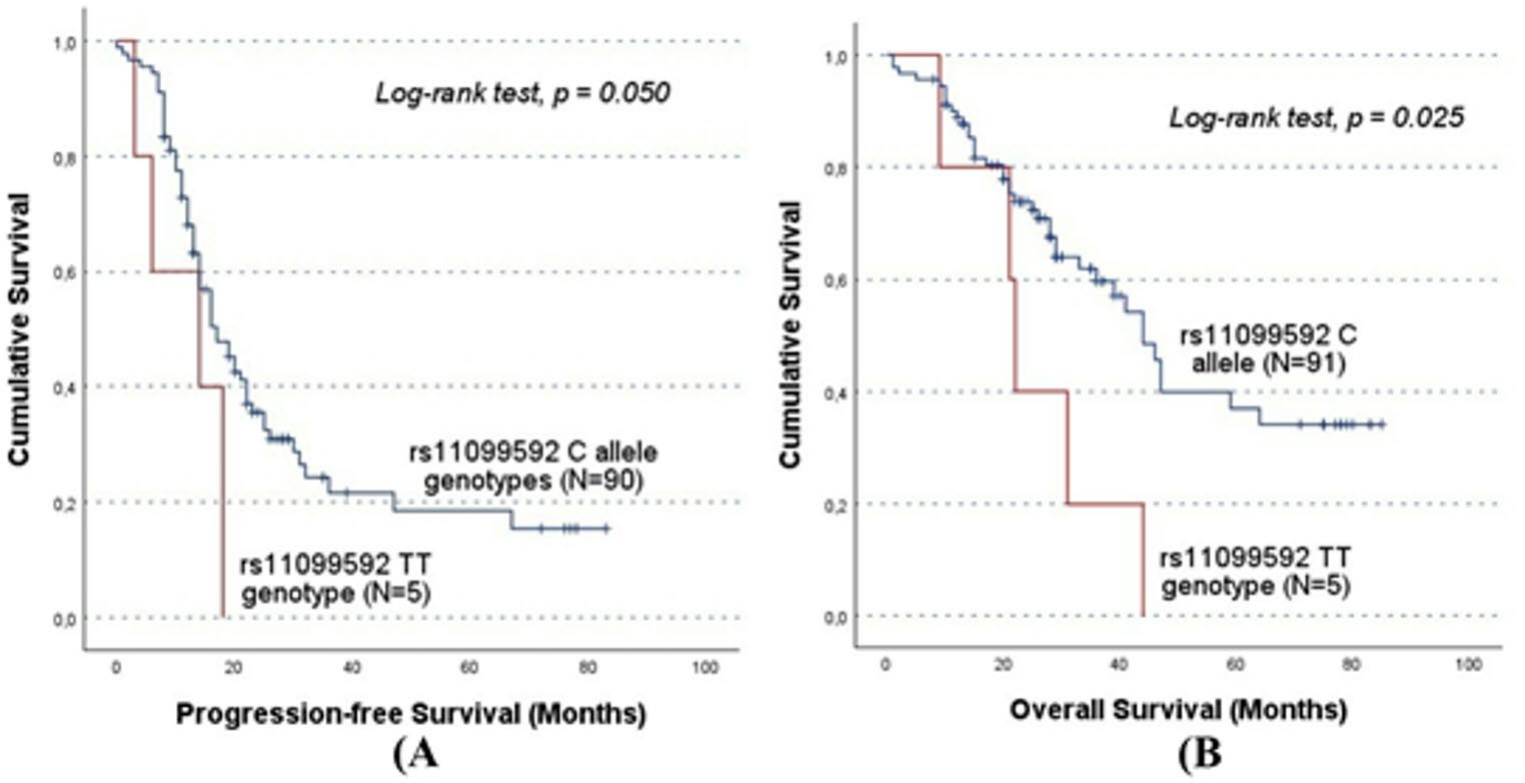
Progression-free survival (PFS) and overall survival (OS) by Kaplan-Meier and log-rank test for OC patients in cohort A, according to *HPSE* rs11099592 genotype distribution. The TT genotype group showed lower survival times compared to C allele carriers. **A**) The mean PFS for C allele carriers was 28.8 ± 3.3 months, while TT carriers presented a mean PFS of 11.8 ± 3.1 months (CC/CT vs. TT; log-rank test, *p* = 0.050). B) The mean OS for C allele carriers was 48.9 ± 3.9 months, while for TT genotype carriers the mean OS was 25.4 ± 5.8 months (CC/CT vs. TT; log-rank test, *p* = 0.025). However, both findings should be evaluated carefully, given the under representation of the TT genotype. The significant results concerning the impact of the SNPs on patients’ prognosis in cohort A were validated in the independent cohort B. While no significant association was detected in the entire cohort B, stratified analyses confirmed the association between the rs4364254 C and rs11099592 T alleles and poorer clinical outcomes. Among the patients resistant to platinum, the rs4364254 Callele was associated with lower OS compared to the TT genotypes (CC/CT vs.TT; mean OS of 19.9 ± 2.4 months and 28.8 ± 3.4 months; log-rank test, p = 0.044; Fig. 4). As for rs11099592, non-serous OC patients carrying the T allele presented a worse OS than their counterparts with the CC genotype (TT/CT vs. CC; mean OS of 45.1 ± 3.3 months and 53.5 ± 1.6 months, respectively; log-rank test, p = 0.016; Fig. 5)

**Fig. 4:**
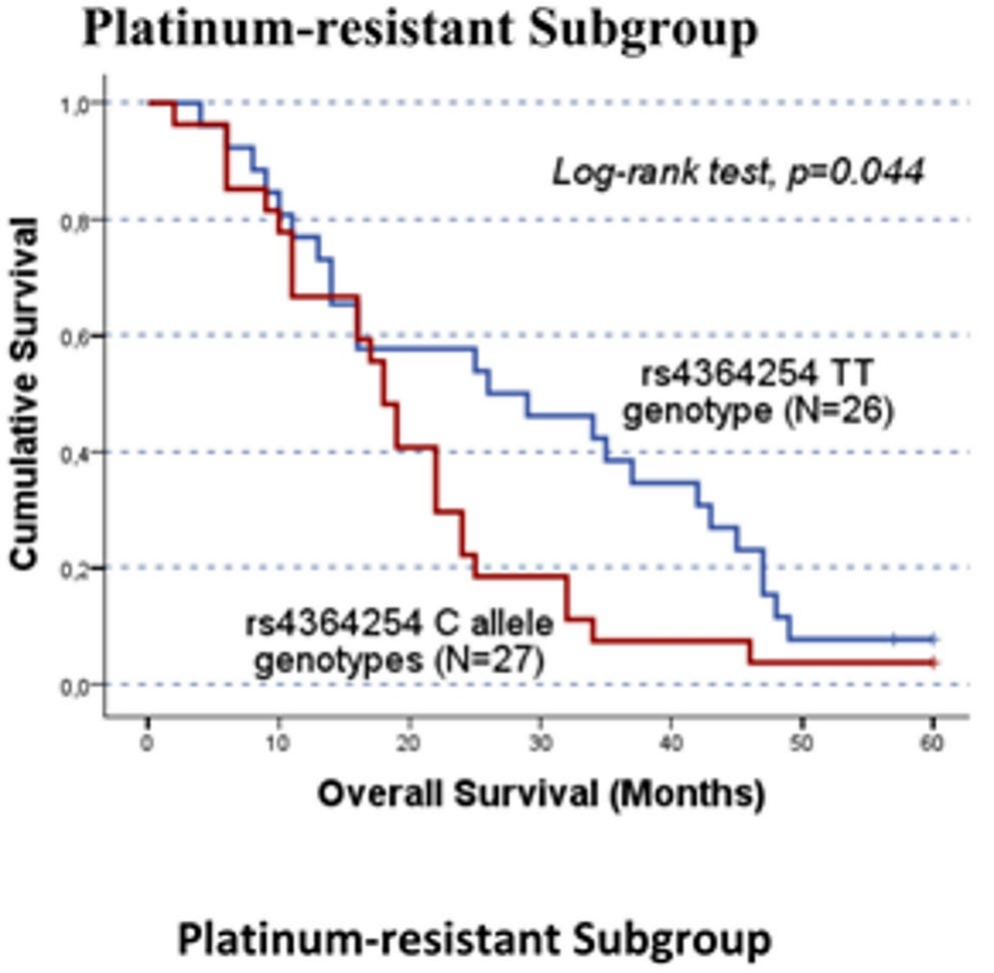
Overall survival (OS) by Kaplan-Meier and log-rank test for cohort B platinum-resistant OC patients, according to *HPSE* rs4364254 genotype distribution. C allele carriers had a worse OS than TT allele carriers (CC/CT vs.TT; log-rank test, *p =* 0.044). The mean OS for C allele and TT genotype carriers was 19.9 ± 2.4 months and 28.8 ± 3.4 months, respectively

**Fig. 5:**
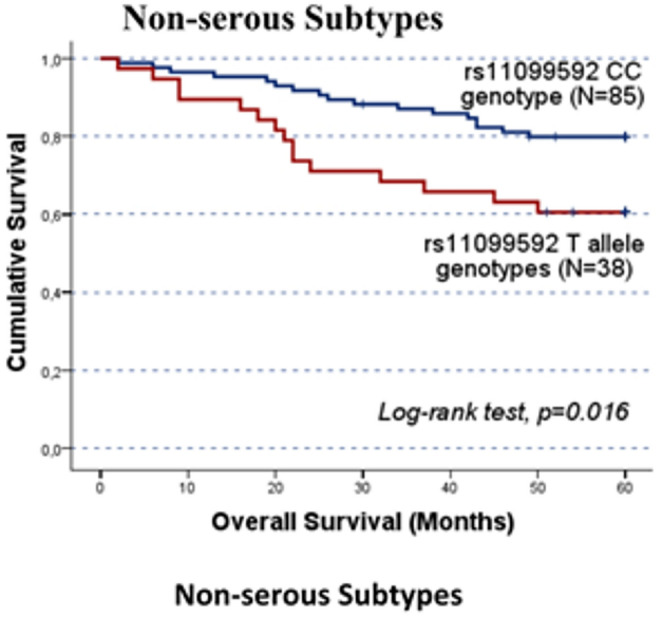
Overall survival (OS) by Kaplan-Meier and log-rank test for cohort B non-serous OC patients, according to genotype distribution for *HPSE* rs11099592. T allele carriers presented a worse OS than CC allele carriers (TT/CT vs. CC; log-rank test, *p =* 0.016). T allele and CC genotype carriers had a mean OS of 45.1 ± 3.3 months and 53.5 ± 1.6 months, respectively

The original article has been corrected.

